# Cholera

**Published:** 1883-07-20

**Authors:** 


### *CHOLERA.*

There is reason to apprehend a visitation of cholera to this country at no distant period. It is now prevailing in Egypt. A writer in Pall Mall Gazette, Dr. B. G. Jenkins—(New York Med. Record). "Dr. Jenkins, who professes to have made a study of cholera, maintains that there are two forms of the disease, one originating in India, and the other in Arabia. It is the Arabian variety, he asserts, that has always heretofore overrun western Europe, and the present outbreak in Egypt is more readily traced to Mecca than to the Ganges. His inference is, that before the close of the year cholera will be raging in every quarter of the globe. Without accepting Dr. Jenkins' theories, the part of wisdom may nevertheless lie in acting as if they were proved.

"Another element of danger is to be found in the fact that an independent focus of infection has come to light. We refer to the prevalence of the disease at Shanghai and Swatow. In view of this fact, the health officers of Atlantic ports will have to keep watch of pretty much our whole commerce, instead of concentrating their attention upon vessels arriving from the Mediterranean, and the authorities on the Pacific coast must share their vigilance."

